# Effect of a breastfeeding educational intervention: a randomized controlled trial*

**DOI:** 10.1590/1518-8345.3081.3335

**Published:** 2020-09-30

**Authors:** Erdnaxela Fernandes do Carmo Souza, Alfredo Almeida Pina-Oliveira, Antonieta Keiko Kakuda Shimo

**Affiliations:** 1 Hospital Samaritano, Unidade Materno-Infantil, São Paulo, SP, Brazil.; 2 Universidade Guarulhos, Guarulhos, SP, Brazil.; 3 Universidade Estadual de Campinas, Campinas, SP, Brazil.

**Keywords:** Postpartum Period, Women’s Health, Breast Feeding, Health Education, Biomedical Technology, Obstetric Nursing, Puerpério, Saúde da Mulher, Aleitamento Materno, Educação em Saúde, Tecnologia Biomédica, Enfermagem Obstétrica, Periodo Posparto, Salud de la Mujer, Lactancia Materna, Educación en Salud, Tecnología Biomédica, Enfermería Obstétrica

## Abstract

**Objective:**

to assess the effect of a breastfeeding educational intervention on the counseling provided to postpartum women.

**Method:**

this is a randomized controlled trial including 104 postpartum women (intervention group = 52 and control group = 52) from a private hospital, whose educational intervention was based on the pragmatic theory and on the use of a soft-hard technology called Breastfeeding Educational Kit (*Kit Educativo para Aleitamento Materno*, KEAM). Women were followed-up for up to 60 days after childbirth. Chi-Squared Test, Fischer’s Exact Test, and Generalized Estimating Equation were used, with a significance level of 5% (p-value <0.05). The analyses were performed using the Statistical Package for the Social Sciences, version 24.

**Results:**

the postpartum women in the intervention group had fewer breastfeeding difficulties and a higher percentage of exclusive breastfeeding at all time points compared with those in the control group.

**Conclusion:**

the educational intervention based on active methodologies and stimulating instructional resources was effective in developing greater practical mastery among postpartum women with regard to adherence and maintenance of exclusive breastfeeding. Registry REBEC RBR – 8p9v7v.

## Abstract (pt)

**Objetivo:**

avaliar o efeito de uma intervenção educativa para aleitamento materno no aconselhamento às puérperas.

**Método:**

trata-se de um ensaio clínico randomizado e controlado com 104 puérperas (grupo intervenção = 52 e grupo controle = 52) de um hospital privado, cuja intervenção educativa foi fundamentada na teoria pragmática e no emprego de tecnologias leve-dura denominada “Kit Educativo para Aleitamento Materno” (KEAM). As puérperas foram monitoradas até 60 dias após o nascimento do bebê. Utilizaram-se o teste de Qui-Quadrado, o Teste Exato de Fischer, a Equação de Estimativa Generalizada e o nível de significância de 5% (p-valor <0.05). As análises foram realizadas no *Statistical Package for the Social Sciences* versão 24.

**Resultados:**

puérperas do grupo intervenção apresentaram menores dificuldades na amamentação e maior percentual de aleitamento materno exclusivo em qualquer tempo observado quando comparado ao grupo controle.

**Conclusão:**

a intervenção educativa baseada em metodologias ativas e recursos instrucionais estimulantes foi efetiva para desenvolver maior domínio prático das puérperas na adesão e na manutenção do aleitamento materno exclusivo. Registro REBEC RBR-8p9v7v.

## Abstract (es)

**Objetivo:**

evaluar el efecto de una intervención educativa sobre lactancia materna en el asesoramiento de mujeres puérperas.

**Método:**

se trata de un ensayo clínico controlado y aleatorizado con 104 puérperas (grupo de intervención = 52 y grupo de control = 52) de un hospital privado, cuya intervención educativa se basó en la teoría pragmática y en el uso de la tecnología blanda-dura llamada “Kit educativo sobre lactancia materna” (KELM). Las puérperas fueron monitoreadas hasta 60 días después del nacimiento del bebé. Se utilizó la prueba de Chi-cuadrado, la prueba exacta de Fischer, la ecuación de estimación generalizada y un nivel de significancia del 5% (valor p<0,05) Los análisis se llevaron a cabo en el *Statistical Package for the Social Sciences* versión 24.

**Resultados:**

las mujeres puérperas del grupo de intervención presentaron menos dificultades para amamantar y un mayor porcentaje de lactancia materna exclusiva en cualquier momento observado en comparación con el grupo de control

**Conclusión:**

la intervención educativa basada en metodologías activas y recursos instructivos estimulantes fue eficaz para desarrollar un mayor dominio práctico por parte de las puérperas en la adopción de la práctica y el hábito de la lactancia materna exclusiva. Registro REBEC RBR-8p9v7v.

## Introduction

The World Health Organization^(^ 1 ^)^ recommends exclusive breastfeeding (EBF) up to six months of the infant’s life and supplemental breastfeeding up to two years of age and beyond, since it is directly related to health promotion and prevention of infant morbidity and mortality. However, many women face difficulties regarding the practical management of breastfeeding and/or associated with external factors, which implies the discontinuation of this protective behavior.

Thus, the implementation of innovative strategies and technological resources in the field of health education may greatly contribute to women’s learning in order to strengthen engagement in preventive behaviors and to promote BF.

In this sense, it is understood that the concept of Health Technologies encompasses any form of intervention used to promote, prevent, diagnose, or treat diseases, as well as to promote rehabilitation or short-, medium-, and long-term care, including devices, procedures, medications, materials, programs, and care protocols, in addition to organizational, educational, information, and support systems, through which health care is provided to the population^(^ 2 ^)^.

Nurses are understood as having a key role in the use of these Health Technologies to achieve the best indicators in BF promotion. This is a dual challenge in facing barriers and in encouraging good BF practices mediated by different scientific knowledge, research methods, and educational processes conducted by the nursing team^(^ 3 ^)^.

To that end, Health Technologies were classified as soft, soft-hard, and hard^(^ 4 ^)^. Soft technology is related to interpersonal relations, welcoming, and creation of bonds. Soft-hard technology is related to well-structured knowledge, such as work process or certain fields of knowledge. Finally, hard technology is characterized by concrete materials, such as machines, equipment, and organizational structures^(^ 4 ^)^.

This study used a soft-hard technology^(^ 4 ^)^ based on John Dewey’s pragmatic theory, with the aim of implementing an educational action focused on learner’s experience and valuation of practices^(^ 5 ^-^ 7 ^)^. This educational approach is known to be related to the evolution of the human mind and of knowledge on certain situations of social life, to the centrality of the individual in distinguishing objects in the long-term memory, and to reflexive positioning about one’s own experienced reality.

The present study aims to fill a gap in new technology-mediated health educational strategies of nursing care provided to postpartum women at hospital discharge with the purpose of encouraging EBF. Thus, the aim of the study was to assess the effect of a BF educational intervention on the counseling provided to postpartum women.

## Method

This is a randomized controlled trial including 104 postpartum women treated in the maternity ward of a private hospital from August 2016 to March 2017.

The Control Group (CG) received routine institutional guidelines on BF by the nursing team, namely: verbal guidance and assistance in the practical management of BF, such as BF positions, correct baby’s latch-on, making the baby burp, provision of on-demand BF, use of lanolin after feedings, and clarification of doubts.

The Intervention Group (IG) underwent an educational intervention based on the pragmatic theory using the soft-hard technology called “*Kit Educativo para Aleitamento Materno*” (KEAM). Dialogical, visual, and interactive approaches for the practical management of BF were valued, with the aim of creating opportunities for pregnant women to manipulate the items included in the KEAM, allowing for practical simulations of use or for the selection of each item, providing instantaneous feedback, and clarifying doubts.

The study included pregnant women whose infants were below 60 days of age, according to the following inclusion criteria: having a landline or cell phone and practicing EBF during hospitalization in the rooming-in facility. The exclusion criteria were the following: medium- and high-risk mothers and infants, or pre-term infants who were not able to be breastfed, as well as postpartum women with communication difficulties, e.g., with a hearing disability or who did not speak Portuguese.

Randomization was performed with numbered, opaque, and sealed envelopes indicating the group to which each woman would be allocated, which were opened by the women themselves or by a companion.

**Figure 1:**
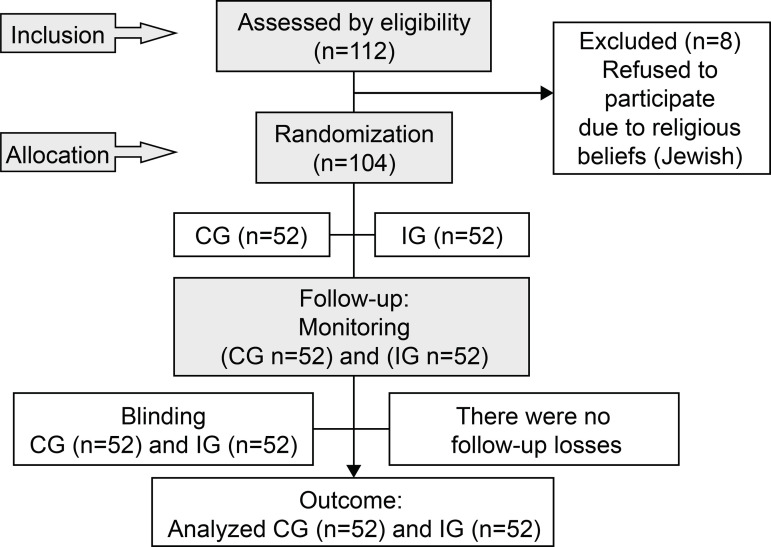
Diagram representing the flow of participants in each stage of the study according to the CONSORT statement for non-pharmacological interventions. São Paulo, SP, Brazil, 2018

The sample size was estimated by a pilot test (52 subjects in each group), totaling 104 participants. The data were described as absolute frequencies and percentages for the qualitative variables, and as position and dispersion measures for the quantitative variables. The details of the investigation process (Figure 1) followed the Consolidated Standards of Reporting Trials (CONSORT) recommendations.

The comparisons of the variables across groups were conducted using the Chi-Square test and, when necessary, Fisher’s exact test, and the comparative analyses of the variables across groups and time points were conducted using the Generalized Estimating Equation model^(^ 8 ^)^. The significance level was set at 5% (p-value <0.05), and the analyses were conducted using the Statistical Package for the Social Sciences (SPSS), version 24.

The development of the KEAM was based on the problems/concerns/difficulties expressed by postpartum women at the time of postpartum monitoring, designed by the lead researcher during the development of her Master’s dissertation^(^ 9 ^)^, such as latch-on difficulties, nipple sensitivity/pain, nipple trauma, believing they have weak milk, absence of breast milk (BM), breast implant, breast engorgement, twin birth, excessive baby crying, baby’s sleepiness, baby’s weight loss, use of silicone nipple, maternal insecurity regarding breastfeeding time, infant reflux, fear of choking, use of the breast as a pacifier, and use of supplementary food.

**Figure 2:**
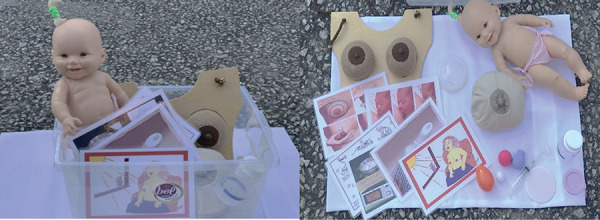
“*Kit Educativo em Aleitamento Materno*” (KEAM) São Paulo, SP, Brazil, 2018

Based on the diagnosis of these problems, the development of the KEAM (Figure 2) focused on the selection of materials available in the market and easy to sanitize, consisting of 15 items identified by the main researcher as strategic instruments to prevent misunderstandings of certain guidelines and/or inadequate execution of medical prescriptions.

(1) Didactic doll: used to teach the practical management of BF, especially to illustrate BF positions, how to place the baby to burp, and how to place them after feedings, in order to minimize difficulties and maternal insecurity. This provides postpartum women with the opportunity of engaging in practical simulations of the provided guidelines with the didactic doll, as well as of clarifying doubts.

(2) Illustrative card on possible BF positions: used as a supplement to item 1 to illustrate the possible adequate positions that may be used during BF.

(3) Illustrative card on correct baby’s latch-on: used to illustrate the correct latch-on of the nipple-areolar area and adequate opening of baby’s mouth to grab most of the areola.

(4) Didactic breast: used to illustrate the internal and external anatomy of the breast, types of nipple, and to teach how to do a circular massage, to extract BM manually, and to use the own mother’s colostrum on the nipples after feedings (used exclusively by the researcher in the demonstration).

(5) Illustrative card on how to do a circular massage and BM extraction: used to display how to do the circular massage and to extract BM, as a supplement to item 4.

(6) Didactic material on the capacity of the baby’s stomach: used to illustrate the capacity of the infant’s stomach at one, three, and seven days of life. It consists of spheres that indicate the amount of milk that may be consumed by the baby over time, because many breastfeeding women feel insecure about this topic.

(7) Protective breast shells: used in the educational intervention to protect sensitive nipples and in the presence of nipple trauma. They are devices made of plastic materials, have the shape of a disc and a round orifice in the center, and are placed inside the bra and over the nipples. They usually have two types of bases: a rigid one and a flexible one with specific indications. Shells with a rigid base are used to help the protrusion of flat nipples during pregnancy; those with a flexible base are commonly used after childbirth as a nipple protector. In this study, shells with a flexible base were indicated for postpartum women presenting sensitive nipples and/or nipple trauma such as bruises and/or fissures. The shells have ventilation orifices that allow for air circulation and prevent the injured nipple-areolar tissue from adhering to the clothes. They are also used to collect milk that leaked during lactation so that it does not wet the mother’s clothes. It is worth noting that the collected milk should be disregarded and that these shells should not be used during sleep, in order to avoid local compression. Furthermore, the importance of constant sanitation of the shells throughout the day was reinforced, preferably after every feeding, thus preventing the proliferation of microorganisms.

(8) *Latch Assist* ^®^ and *Niplette* ^®^: used to assist/stimulate protrusion of flat and/or inverted nipples, when latch-on difficulties were caused by these types of nipple.

(9) Breast gel protector: the educational intervention provided guidance on its correct use, sanitation after use, and proper storage in the fridge. It is a protective disc that absorbs the liquid excess and is commonly used to treat nipple trauma and sensitivity. There are recommendations to use it cooled so as to provide relief. Its time of use ranges from three to seven days, and it should be constantly sanitized.

(10) Illustrative card on 100% purified lanolin: used to provide instructions on its use in cases of nipple sensitivity/trauma, after feedings, since it is routinely prescribed. This natural wax obtained by boiling sheep wool in water is highly purified, hypoallergenic, tasteless, odorless, and it has low levels of pesticides, being indicated in the prevention and treatment of nipple trauma.

(11) Illustrative card on the importance of exposing the breasts to a sunbath: used to inform about the beneficial effects of sun rays on trauma healing, in addition to stimulating the production of vitamin D, which strengthens the skin. Women were instructed to expose their breasts to sun rays for at least 10 minutes and for a maximum of 30 minutes a day, before 10 a.m. or after 4 p.m.

(12) Illustrative card on manual and mechanical extraction of BM: used to display the possible ways of extracting BM for storage if necessary and the required precautions: performing the extraction in a clean place; washing hands properly; keeping hair tied up; preferably using a mask but, if not possible, avoid speaking during the procedure.

(13) Container for storing BM: women were presented the container for storing the extracted milk, which may be made of glass or of transparent plastic with a plastic cap and is easily available in the market. Explanations were given about how to sanitize the container by rinsing it in running water with neutral soap, boiling the cap and the vial for 15 to 20 minutes, leaving it to dry over a clean cloth, and then keeping it in a tightly closed container. The maximum storage time was established at 12 hours in the first shelf of the fridge and at 15 days in the freezer. It was recommended to identify each vial with date and time of collection and to store in each vial only the approximate volume required for each meal of the baby, according to medical recommendations.

(14) Cup for the provision of BM: it was presented as an option to provide the extracted BM when the mother is absent/unavailable. There was a demonstration of the technique for this procedure (placing the baby, who should be awake and calm, on the lap, in the seated or semi-seated position, touching the cup brim on the baby’s lower lip, and letting the milk touch the lips of the baby, who will make movements to “lick” the milk and then to swallow it).

(15) Illustrative card on how to store, prepare, and provide BM in the cup (if necessary and after the mother’s return to work): used as a supplemental material for items 13 and 14 to illustrate how to store the milk (fridge/freezer), to defrost it in warm water (“Bain-Marie”), emphasizing that BM should not be boiled or heated in the microwave, and to provide BM in the cup.

The data were collected at two different stages: in the first stage, the procedure was carried out by the main researcher during postpartum at the maternity ward, from 24 to 72 hours after childbirth. In the second stage, after the women’s hospital discharge, another professional trained by the researcher performed the blinding, monitoring data from the two groups on the following: type of breastfeeding, difficulties found during breastfeeding, and whether the mother was receiving support at home and, if she was, what and from who was this support, at three different time points (days 10, 30 and 60). This researcher was blind as to which group they belonged, thus preventing possible biases that could arise from a previous contact with the researcher.

The study was approved by the Ethics and Research Committee of the Samaritano Hospital of São Paulo, under the no. 1.946.830 and under the registry RBR-8p9v7v in the Brazilian Registry of Clinical Trials.

## Results

The study included 104 postpartum women from a private health institution in the state of São Paulo, Brazil, of whom 52 were allocated to the IG and 52 to the CG. The women in the IG showed a higher percentage of EBF compared to those in the CG at all time points, with statistical significance (p<0.05). At day 10, there was a higher percentage of EBF than at days 30 or 60 (Table 1).

**Table 1:** Follow-up of the breastfeeding practice after hospital discharge. São Paulo, SP, Brazil, 2018

	Day 10	Day 30	Day 60
**Breastfeeding - CG** *			
EBF^†^	37(71.2%)	25(48.1%)	23(44.2%)
NEBF^‡^	11(21.2%)	18(34.6%)	12(23.1%)
FF^§^	4(7.7%)	9(17.3%)	17(32.7%)
**Breastfeeding - IG** ^||^			
EBF^†^	48(92.3%)	42(80.8%)	45(86.5%)
NEBF^‡^	4(7.7%)	10(19.2%)	6(11.5%)
FF^§^	0(0%)	0(0%)	1(1.9%)
**Breastfeeding-Total**			
EBF^†^	85(81.7%)	67(64.4%)	68(65.4%)
NEBF^‡^	15(14.4%)	28(26.9%)	18(17.3%)
FF^§^	4(3.8%)	9(8.7%)	18(17.3%)
**p-value** ^¶^	**Group**	**Time**	**Group x Time**
	<0.0001	<0.0001	0.1188

* CG = Control group;

† EBF = Exclusive breastfeeding;

‡ NEBF = Non-exclusive breastfeeding;

§ FF = Formula feeding;

|| IG = Intervention group;

¶ GEE = (Generalized Estimating Equation)

There was statistical significance with regard to maintaining BF for a longer time and a lower percentage of difficulties at the study time points in the group that underwent the intervention with the KEAM in the maternity ward before hospital discharge compared with the CG (Table 2).

**Table 2:** Follow-up of the breastfeeding difficulties after hospital discharge. São Paulo, SP, Brazil, 2018

		Breastfeeding			p-value
		EBF*	NEBF^†^	FF^‡^	
Day 10	Difficulty - CG^§^				
	No	26(100%)	0(0%)	0(0%)	<0.0001^||^
	Yes	11(42.3%)	11(42.3%)	4(15.4%)	
	Difficulty - IG^¶^				
	No	40(100%)	0(0%)	0(0%)	<0.0001**
	Yes	8(66.7%)	4(33.3%)	0(0%)	
	Difficulty-Total				
	No	66(100%)	0(0%)	0(0%)	<0.0001^||^
	Yes	19(50%)	15(39.5%)	4(10.5%)	
Day 30	Difficulty - CG^§^				
	No	19(100%)	0(0%)	0(0%)	<0.0001^††^
	Difficulty - IG^¶^				
	No	39(100%)	0(0%)	0(0%)	<0.0001^††^
	Yes	3(23.1%)	10(76.9%)	0(0%)	
	Difficulty-Total				
	No	58(100%)	0(0%)	0(0%)	<0.0001^||^
	Yes	9(19.6%)	28(60.9%)	9(19.6%)	
Day 60	Difficulty - CG^§^				
	No	21(100%)	0(0%)	0(0%)	<0.0001^††^
	Yes	2(6.5%)	12(38.7%)	17(54.8%)	
	Difficulty - IG^¶^				
	No	43(100%)	0(0%)	0(0%)	<0.0001^††^
	Yes	2(22.2%)	6(66.7%)	1(11.1%)	
	Difficulty-Total				
	No	64(100%)	0(0%)	0(0%)	<0.0001^||^
	Yes	4(10%)	18(45%)	18(45%)	

* EBF = Exclusive breastfeeding;

† NEBF = Non-exclusive breastfeeding;

‡ FF = Formula feeding;

§ CG = Control group;

|| Likelihood Ratio Test;

¶ IG = Intervention group;

** Fisher's Exact Test;

†† Chi-Square Test

Among the difficulties reported by the postpartum women, the most prevalent across all time points (days 10, 30 and 60 of the baby’s life) in the CG were the following: reduced BM production, baby’s weight loss, latch-on difficulties, breast fissure, mastitis, excessive baby’s crying, maternal insecurity (believing they have weak milk), impression that the BM had dried up.

Conversely, the most prevalent difficulties in the IG were the following: baby’s weight loss, excessive baby’s crying, reduced BM production, baby’s sleepiness, guidance, and medical prescription of supplementation. However, a statistical significance (p<0.05) was observed in the IG with regard to whether having difficulties or not compared to the CG at all time points.

## Discussion

Although EBF rates have increased in Brazil, it is observed that they are still much lower than the recommended ones in the last three decades^(^ 10 ^)^. It is known that the BF practice is possible for almost all mothers, but there are several difficulties that contribute to early BF discontinuation^(^ 11 ^)^.

Among the most prevalent difficulties found in the present study, most were similar in both groups, but there was a statistically significant lower percentage of difficulties in the IG at the analyzed time points compared to the CG, which may have some influence on the maintenance of EBF. Several studies^(^ 12 ^-^ 15 ^)^ corroborate these findings, reporting the same barriers to the BF practice.

A number of studies on the prevalence of BF among users of private health care services are still scarce, but the results of this study reveal that the prevalence of EBF at 60 days of the baby’s life was 65.4%, a percentage that was a little lower than the one observed in a similar study, which found a prevalence of 79.0% at the same time point^(^ 13 ^)^.

Statistically significant results were also observed in the IG, where the participants received an education intervention during hospitalization in the maternity ward, using a soft-hard technology to receive verbal and visual guidance on BF. This group was able to minimize difficulties and to maintain a greater percentage of EBF at any analyzed time point compared with the CG, which only received routine guidelines by the nursing team of the study site.

The nurses’ knowledge on the clinical management of BF are of paramount importance to favor guidance strategies, but these professionals stated being more successful when verbal guidelines are combined with visual instruments which, in turn, are not always available^(^ 16 ^)^.

It is increasingly evident that there is a need for implementing the use of didactic materials and devices capable of assisting and reinforcing the guidelines provided by Nursing and/or health care professionals on the practical management of BF in health institutions, in view of the significant results shown in the present study.

Another key aspect of this study was the fact the educational intervention was based on the pragmatic theory, which provided postpartum women with an opportunity to have a practical experience mediated by the KEAM so that they could manipulate the didactic materials, value illustrative items, and clarify doubts, in order to improve control over their difficulties, desires, and strengths.

Other studies^(^ 17 ^-^ 25 ^)^ that used technologies as health education strategies reinforce evidence of innovations in BF assistance, such as: use of educational games, electronic media, educational manuals, information booklets, video conferencing, and digital instant messaging. These practices showed to be effective in adherence and maintenance of breastfeeding supported by educational technologies based on greater interaction and protagonism of the participating women.

One limitation of this study is the low number of childbirths at the institution where the study was carried out; thus, it is necessary to expand the period of data collection and follow-up in order to reach the proposed sample size.

Innovations in health education actions to improve adherence and maintenance of BF for a longer time are a technical and ethical imperative to overcome several barriers to this good practice in the health context of mothers and children, without disregarding their families and their community resources.

Thus, the present study contributed to the Nursing educational practices, because it reinforces the importance of using educational materials that are potentially significant to promote BF in health institutions and to value the use of active methodologies based on the theoretical background provided by the pragmatic theory for the systematization of educational interventions.

## Conclusion

This study showed the effectiveness of an educational intervention in the counseling of postpartum women mediated by concrete and manipulable educational technologies gathered in the KEAM, since this soft-hard technology provides verbal, visual, and tactile stimulation in a dialogical and inter-subjective context that positively influences learning by creating practical experiences about BF.
